# The seven phases of match status differentiate the running performance of soccer players in UEFA Champions League

**DOI:** 10.1038/s41598-023-33910-9

**Published:** 2023-04-24

**Authors:** Marek Konefał, Łukasz Radzimiński, Jan Chmura, Toni Modrić, Michał Zacharko, Alexis Padrón-Cabo, Damir Sekulic, Sime Versic, Paweł Chmura

**Affiliations:** 1 Department of Human Motor Skills, Wrocław University of Health and Sport Sciences, Paderewskiego 35, 51-612 Wrocław, Poland; 2 Department of Physiology, Gdansk University of Physical Education and Sport, 80-336 Gdansk, Poland; 3 Faculty of Kinesiology, University of Split, 21 000 Split, Croatia; 4 Department of Physical Education and Sport Science, University of A Coruña, 15071 A Coruña, Spain; 5 HNK Hajduk, Split, Croatia; 6 Department of Team Games, Wrocław University of Health and Sport Sciences, Wrocław, Poland

**Keywords:** Human behaviour, Physiology

## Abstract

The purpose of this research was to investigate the running performance of professional soccer players in relation to seven phases which resulted in the changing or maintaining the match status in the UEFA Champion League games during season 2020/2021. Moreover, we aimed to define which match status phases occur at the earliest stage of regular game time. This study involved professional soccer players from 24 teams participating in the group stage of UEFA Champions League in season 2020/21. The match status was divided into seven phases that result in changing or maintaining the match outcome: DW (Drawing to Winning); LD (Losing to Drawing); WW (Winning to Winning); DD (Drawing to Drawing); LL (Losing to Losing); DL (Drawing to Losing); WD (Winning to Drawing). Such running performance variables as: total distance covered (TDC) and distance covered in high-intensity running (HIR) were analyzed. Players participating in the UEFA Champions League matches cover the longest TDC in DW, DL and DD phases. TDC in these stages was between 111 and 123 m min^−1^. The highest HIR was recorded during phases: DW, DL and LL (range between 9.91 and 10.82 m min^−1^). In contrast, the lowest total distance and distance in HIR is covered during WD phase (only 105.57 ± 1.89 m min^−1^ and 7.34 m min^−1^ respectively). On average, phases resulting in the change of the match status occur during the first half, while all phases maintaining the result in the second half. Coaching staffs should consider registering and analysing the physical match performance in relation to described seven match status phases. Such information allows to prepare team-specific training drills, that players should perform more often in order to change or maintain the status of the game.

## Introduction

In professional soccer competitions such as UEFA Champions League, the main purpose for the teams is to achieve positive result^1^. Thus, the most important situational variable in professional soccer analysis is match outcome (win, draw or lose)^2,3^. However, if the game result will be analyzed through the score line, available literature provides references to the momentary result of the match—so called match status (winning, drawing, losing)^4^. Previously Konefał et al.^5^ reported that scoring or losing a goal creates the possibility to divide the match status into seven different phases. These phases are related to changing or maintaining the match status. Starting with the most favorable situations in terms of competition regulations (won game—three points, draw—one point, lost game—zero points), there is a possibility to increase the number of collected points when the phase of game is changing from the drawing to winning (DW)—from one point to three points for the victory. Further, there is a phase from losing to drawing (LD), where number of collected points change from zero to one. Moreover, there are phases related to maintaining the match status: continuation of winning (WW), drawing (DD) or losing (LL). Finally, the match status changes may result in losing the points when phase changes from drawing to losing (DL)—loss of one point, or from winning to drawing (WD)—loss of two points.

Soccer is a low-scoring sport, therefore every scored or lost goal influences the game^6,7^. Thus, changes in match status may result in changes in players and teams match performance. These changes affect tactical, technical and physical performance of the players during the match^8–10^. It was previously demonstrated that professional soccer players perform less high-intensity actions when winning than when drawing or losing^10–14^. Furthermore, when the team is losing, players through the higher activity make an effort to change the negative result into a draw and then into a win^15^. In contrast, some authors reported that teams do not change their performance according to the match status^16^.

Most of the distance during the soccer match is covered in walking, jogging or running with low intensity^3^. Therefore, the level of aerobic capacity is one of the crucial fitness components for the players^17^. Total distance covered by the players (TDC) during the 90-min game is usually between 9000 and 12,500 m (100–130 m min^−1^)^18,19^. It should be emphasized that in modern soccer high-intensity actions often plays the crucial role in the goal scoring actions^20^. Such activities as accelerations, decelerations, distance covered in high-intensity running (speed > 19.8 km h^−1^, HIR) and number of sprints are worth to mention ^21,22^. Distance covered in high intensity during the game exceed even 1800 m (6–8 m·min^−1^)^3,23^, however, it is dependent on the assumed speed thresholds^24,25^ and tactical position of the players^24^. The longest total distance is usually covered by the central midfielders, while external defenders and external midfielders cover the longest distance in HIR^26^.

Knowledge about how professional soccer players react on the changing or maintaining match status could provide numerous practical information for coaches about preparing their teams for such situations^16^. Although there are several studies describing the running match performance in relation to match status, it is still unknown how does the physical performance change during the phases when the teams score or lose a goal. Minute of the game when the first goal is scored/lost seems to be important in relation to the players activity as well. Therefore, the purpose of this research was to investigate the running performance of professional soccer players in relation to seven phases which resulted in the changing or maintaining the match status in the UEFA Champion League games during season 2020/2021. Moreover, the current study aimed to define which match status phases occur at the earliest stage of regular game time. It was hypothesized, that running performance of professional soccer players will be different in seven phases match status.

## Methods

### Sample

This observational study involved professional soccer players from 24 team participating in the group stage of UEFA Champions League in season 2020/21. The data was obtained from 20 randomly selected matches played in groups A (n = 3), B (n = 3), C (n = 4), E (n = 4), F (n = 3) and G (n = 3). Only field players (goalkeepers were excluded) who played full 90 min were included in the research. Participants were divided into 5 playing positions: central defenders (CD; n = 53), full-backs (FB; n = 46), central midfielders (CM; n = 42), wide midfielders (WM; n = 25) and forwards (FW; n = 16).

To ensure the anonymity of the players all the data were anonymized in accordance with the Declaration of Helsinki. Moreover, this study was approved by the Senate Committee on Ethics of Scientific Research at the Academy of Physical Education in Wroclaw (reference number: 12/2021).

### Procedures

In order to reach all the aims of the current study, the match status was divided into seven phases that result in changing or maintaining the match outcome^5^: DW (Drawing to Winning; 129 observations), when one of the drawing teams score a goal and change the status into winning; LD (Losing to Drawing; 61 observations), when losing team score a goal and the status is changed into drawing; WW (Winning to Winning; 79 observations), when winning team maintain the wining status of the match; DD (Drawing to Drawing; 79 observations), when drawing teams maintain this status; LL (Losing to Losing; 82 observations), when losing team maintain the losing status; DL (Drawing to Losing; 134 observations), when drawing teams lose a goal and the match status change into losing; WD (Winning to Drawing; 62 observations), when winning team lose a goal and game status change into drawing.

The number of minutes throughout the game for each of the aforementioned match status phases varied: DW—33.24 ± 25.27; LD—23.13 ± 18.88; WW—41.87 ± 30.88; DD—61.77 ± 33.75; LL—43.39 ± 30.24; DL—32.19 ± 24.67; WD—25.48 ± 19.32. Therefore, analysed running performance (TDC, HIR—speed > 19.8 km h^−1^)^27^ was presented relatively to playing time (in m min^−1^). Furthermore, the average time of occurrence of each phase was determined by minutes of the match, when goals were scored (e.g., when phase lasted from 10 to 30 min of the game, the phase occurred average in 20th min). Due to the fact, that current study design is based on the individual players observations, the duration and occurrence of each phase was calculated not for the all team, but for each player separately.

The running match performance was recorded and analyzed with a semiautomatic camera tracking system (InStat Fitness, Instat Limited, Limerick, Republic of Ireland). This system is based on the static cameras installed on the soccer stadium crown. Cameras were registering the running match performance of the players (TDC and distance covered in different speed zones) in the real time. Moreover, this system was described in details in previous research^28,29^. The reliability of the InStat Fitness was confirmed by the official testing protocol of Fédération Internationale de Football Association (FIFA) for electronic tracking systems (EPTS).

### Statistical methods

The data were presented as means and standard errors (SE, used to compare the differences between the data) or standard deviations (SD, used when data was summarized). All analysis were conducted using the statistical software R version 4.2.1^30^. Linear mixed models were fitted using the R packages “lme4” and “lmerTest”, adjusted means were calculated using the “emmeans” package, Cohen’s f^2^ was calculated using “multilevelTools” package. Linear mixed models were fitted using the R packages “lme4” and “lmerTest”, adjusted means were calculated using the “emmeans” package. To analyse the dependency between the players’ physical performance and match phases, linear mixed models were used. Phase, Position and Minutes were modelled as fixed effects. Player and match identity were modelled as random effects to account for the repeated measurements. Consequently, the following model structures were fitted:

- $$ {\text{HIR}}\sim {\text{Position}} + {\text{Phase}} + \left( {{1}|{\text{playerID}}} \right) + \left( {{1}|{\text{Match}}} \right) $$
- $$ {\text{TDC}}\sim {\text{Minutes}} + {\text{Position}} + {\text{Phase}} + \left( {{1}|{\text{playerID}}} \right) + \left( {{1}|{\text{Match}}} \right) $$

The significance of the “Phase” variable was verified using the chi-squared test (models with and without this variable were compared). Based on such models, adjusted means for each phase were calculated. Post-hoc comparisons were conducted using the Kenward-Roger’s test and Tukey adjustment. Effect size for “Phase” was calculated as partial Cohen’s f^2^, in two versions: conditional (f^2^_c_) and marginal (f^2^_m_)^31^. Effect sizes for post-hoc comparisons were calculated as Cohen's d. [small (d = 0.2), medium (d = 0.5), and large (d = 0.8)]. The significance level was 0.05.

## Results

Statistical analysis of running match performance in relation to position of soccer players on the field (CD, FB, CM, WM, FW) revealed significant effect in relation to TDC (${\chi }^{2}\left(6\right)=93.2$, $p<0.001$) and HIR (${\chi }^{2}\left(6\right)=71.4$, $p<0.001$)—Table 1.

**Table 1:** The relation between running match performance and position of soccer players on the field in 20 UEFA Champions League matches (mean ± SE).

Position	TDC	HIR
CD; n = 53	105.77 ± 1.46	6.76 ± 0.39
FB; n = 46	113.35 ± 1.54	10.37 ± 0.42
CM; n = 42	122.19 ± 1.62	9.57 ± 0.45
WM; n = 25	115.21 ± 1.95	11.44 ± 0.55
FW; n = 16	108.16 ± 2.30	9.39 ± 0.67

TDC—total distance covered; HIR—distance covered in high-intensity running (speed > 19.8 km h^−1^).

**Figure 1:**
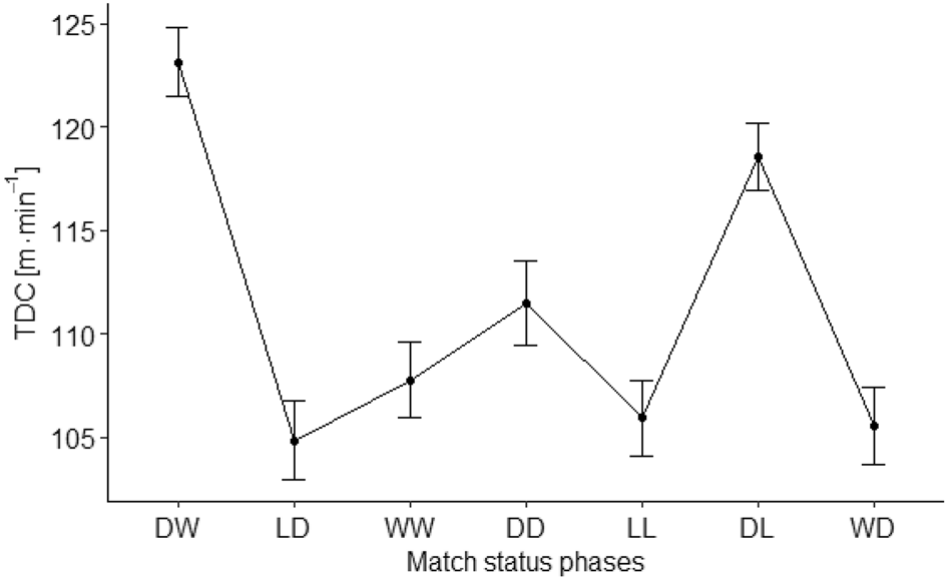
Total distance covered (TDC) on seven match status phases (mean ± SE). DW (Drawing to Winning); LD (Losing to Drawing); WW (Winning to Winning); DD (Drawing to Drawing); LL (Losing to Losing); DL (Drawing to Losing); WD (Winning to Drawing).

Statistical analysis of TDC in relation to match status phases (DW, LD, WW, DD, LL, DL, WD) revealed significant effect ${\chi }^{2}\left(6\right)=152$, $p<0.001$, f^2^_c_ = 0.33, f^2^_m_ = 0.22. Statistically significant differences between match status phases: DW > DL (*p* = *0.016; ES* = *1.36; CI 0.51 to 8.61*), LD (*p* = *0.001; ES* = *5.44; CI 12.83 to 23.71*), WW (*p* = *0.001; ES* = *4.57; CI 9.64 to 21.09*), DD (*p* = *0.001; ES* = *3.47; CI 4.70 to 18.59*), LL (*p* = *0.001; ES* = *5.11; CI 11.25 to 23.11*), WD (*p* = *0.001; ES* = *5.23; CI 12.18 to 22.95*); DL > LD (*p* = *0.001; ES* = *4.08; CI 8.47 to 18.96*), WW (*p* = *0.001; ES* = *3.22; CI 5.04 to 16.58*), DD (*p* = *0.037; ES* = *2.11; CI 0.24 to 13.94*), LL (*p* = *0.001; ES* = *3.76; CI 6.89 to 18.36*), WD (*p* = *0.001; ES* = *3.87; CI 7.70 to 18.31*); DD > LD (*p* = *0.048; ES* = *1.97; CI 0.03 to 13.21*)—Fig. 1 and Table 2.

**Table 2:** The relation between TDC [m·min^-1^], HIR [m·min^-1^] and match status phases (mean ± SE).

	Match status phases						
	DW	LD	WW	DD	LL	DL	WD
TDC	123.14 ± 1.68	104.87 ± 1.90	107.77 ± 1.82	111.49 ± 2.06	105.95 ± 1.83	118.58 ± 1.64	105.57 ± 1.89
HIR	9.91 ± 0.46	8.43 ± 0.58	8.98 ± 0.53	8.56 ± 0.58	10.36 ± 0.53	10.82 ± 0.46	7.34 ± 0.58

TDC—total distance covered; HIR—distance covered in high-intensity running (speed > 19.8 km h^−1^).

DW (Drawing to Winning); LD (Losing to Drawing); WW (Winning to Winning); DD (Drawing to Drawing); LL (Losing to Losing); DL (Drawing to Losing); WD (Winning to Drawing).

**Figure 2:**
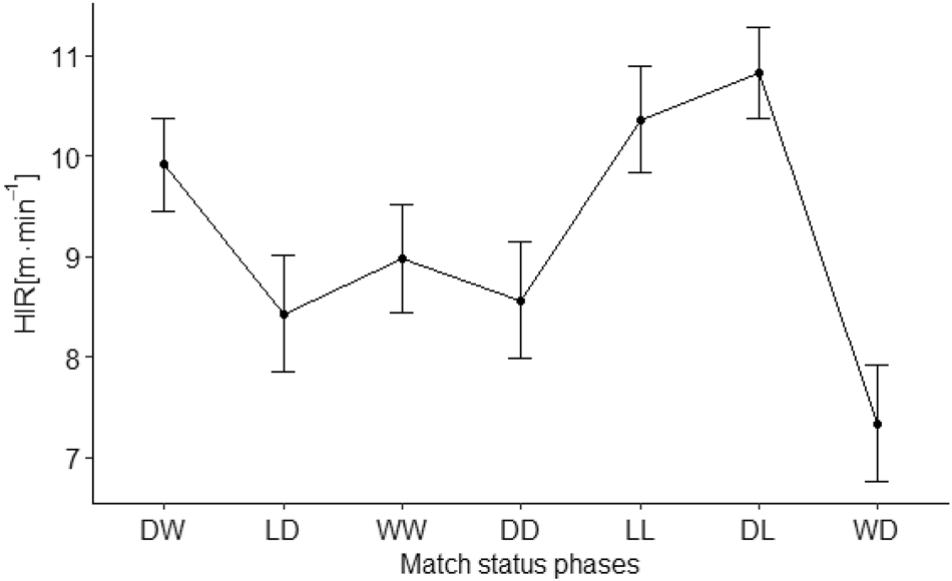
Distance covered at speeds over 19.8 km h^−1^ (HIR) on seven match status phases (mean ± SE). DW (Drawing to Winning); LD (Losing to Drawing); WW (Winning to Winning); DD (Drawing to Drawing); LL (Losing to Losing); DL (Drawing to Losing); WD (Winning to Drawing).

Statistical analysis of HIR in relation to match status phases (DW, LD, WW, DD, LL, DL, WD) revealed significant effect (${\chi }^{2}\left(6\right)=50.3$, $p<0.001$, f^2^_c_ = 0.19, f^2^_m_ = 0.07). Statistically significant differences between match status phases: DL > LD (*p* = *0.001; ES* = *0.71; CI 0.78 to 4.01*), WW (*p* = *0.006; ES* = *0.55; CI 0.33 to 3.36*), DD (*p* = *0.002; ES* = *0.67; CI 0.53 to 3.99*) and WD (*p* = *0.001; ES* = *1.04; CI 1.86 to 5.11*); DW > WD (*p* = *0.001; ES* = *0.77; CI 0.97 to 4.18*); LL > LD (*p* = *0.041; ES* = *0.57; CI 0.04 to 3.82*) and WD (*p* = *0.001; ES* = *0.90; CI 1.16 to 4.89*) —Fig. 2 and Table 2.

**Figure 3:**
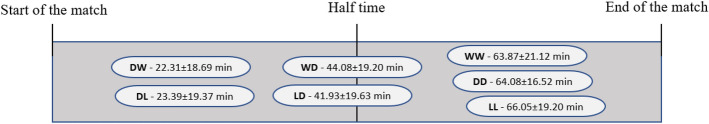
Average minute of the match, when each of the phases occurred (mean ± SD). DW (Drawing to Winning); LD (Losing to Drawing); WW (Winning to Winning); DD (Drawing to Drawing); LL (Losing to Losing); DL (Drawing to Losing); WD (Winning to Drawing).

Different match status phases occur in different stages of the game. Figure 3 shows average minute of the match together with SD, when the concrete phase lasted (calculated on the basis of the minutes, when the goals were scored).

## Discussion

The novel conception of seven different match status phases in relation to physical match performance of professional soccer players was proposed in the current research. The aim of this study was to evaluate players’ running performance during the UEFA Champions League group stage games with reference to seven phases which resulted in changing or maintaining the match status.

The results presented in this investigation demonstrated the UEFA Champions League players cover the longest TDC and distance in HIR during the DW and DL phases. Furthermore, reported relations of the analysed parameters are in line with Goncalves et al.^32^, who calculated correlations between TDC and HIR distance. Nevertheless, the match status phases were not included in their study. Moreover, current research results correspond with data presented by Lago et al.^11^ and Moalla et al.^12^, who emphasized that most intensive stage of the game occur when the team is losing or drawing. In this context it is worth to mention that our DW and DL phases start with drawing. This status is very important, because it occurs at the beginning of the match. Thus, every team is motivated to dominate the opponent and under pressure of the unknown final game result^9^. Moreover, this research demonstrated that the highest activity is registered during phases DW and DL which occur about 22–23 min of the game on average. Akubat et al.^33^ exhibited that players and training staff have to decide how and when invest the energy to reach the best level of performance. The variability of the players’ physical match performance is related to metabolic energy expenditure and increasing fatigue^34,35^. In this context Oliva-Lozano et al.^36,37^ reported that the longest total distance and high metabolic load distance is covered by the players at the beginning of the first half. All the above information could explain observed high levels of physical performance during the phases starting from drawing.

Another interesting and surprising observation was that the large running match performance in HIR was recorded in the phase LL. Due to the fact, that this is the first study describing the seven phases of match status in relation to players running match performance, the traditional concept involving three phases (winning, drawing and losing)^5^ should be used in order to explain our observations. Redwood-Brown et al.^38^ found that initial reaction of the losing teams could increase their physical performance to provide positive changes in the game result. However, if the match status improvement will not occur shortly, the decrease in their intensity is usually observed. Moreover, Fernandez-Navarro et al.^39^ claimed that losing teams increase the frequency of their attacks, what could possibly result in match intensity improvement. Furthermore, the LL phase is one that occur at the lates stage of the match—on average, about 66 min of the game. It can be assumed that although losing teams increase their intensity, the relatively short time left to the end of the match make the change of the adverse result difficult.

The lowest running match performance of the players according to both TDC and HIR distance was noted during the WD phase. Winning status is a comfortable state, thus players could adopt the strategy of keeping the ball and allowing down the pace of the game. One of the possible explanations was introduced by Lago-Peñas^40^ who established that players manage their running match performance and not always use their maximal physical potential. In addition, the reduction of their physical performance after scoring a goal and lost the initiative, could result in allowing the opponents to get back in the match and to score an equalizing goal^38^. In the current research, the results revealed that during the WD phase players covered 105.57 ± 1.89 m·min^−1^ in total and 7.34 ± 0.58 m·min^−1^ in HIR. These values are relatively low in comparison with other phases of match status registered in our study (e.g., in DW phase values of TDC and HIR were 123.14 ± 1.68 m·min^−1^ and 9.91 ± 0.46 m·min^−1^, respectively). Other investigations demonstrated that running match performance differs depending on level of competition or tournament^19,25,41^. To complete the discussion about low running performance in the WD phase, it is worth mentioning that the only study that described 7 phases of match status so far^5^. Specifically, this research analysed the technical performance and found, that in the same WD phase the values of ball possession, number of shots, shots on target, and percentage of accurate passes were the lowest as well. Reduced level of both physical (TDC and HIR distance) and technical performance in this WD phase seem to result in a losing a goal and changing the positive match status.

The conclusions of our research should be interpreted with caution due to some methodological limitations. Concretely, the small sample size could be considered a limitation. However, this sample is composed by elite players who participate in the most important European competition. In addition, further investigations should concern additional contextual variables (e.g. level of opponent) or other measures (e.g. sprinting distance, number of accelerations and decelerations). Moreover, simultaneous analysis of the technical and physical performance in relation to tactical tasks should be taken into consideration as well. Such topic could allow for further exploration of players’ behavior with reference to changing score line.

## Conclusions

Players participating in the UEFA Champions League matches cover the longest TDC in DW, DL and DD phases. TDC in these stages was between 111 and 123 m·min^−1^. Phases DW, DL and DD are characterized by an initial draw, which motivates teams to gain an advantage over the opponent through high level of physical performance.

Regarding the HIR it can be stated that the highest activity was recorded during phases: DW, DL and additionally LL (range between 9.91 and 10.82 m min^−1^). The LL phase occurs at the latest stage of the match (about 66 min of the game on average). Thus, it can be assumed that despite team efforts and high intensity of the game, the unprofitable result is not being changed mostly due to the shortage of time till the end of the game. In contrast, the lowest total distance and distance in HIR is covered during WD phase (only 105.57 ± 1.89 m min^−1^ and 7.34 m min^−1^ respectively). Decrease in the game intensity registered in this phase seem to result in losing a goal and negative change of the match status.

Match status phases analysed in the current research occur in different periods of regular match time. Current study exhibited that DL and DW phases appear at the beginning of the game, LD and WD phases at the end of the first half, while DD, LL and WW phases in the second half. It follows that on average, phases resulting in the change of the match status occur during the first half, while all phases maintaining the result in the second half.

### Practical application

Coaching staffs should consider registering and analysing the physical match performance in relation to described 7 match status phases. Such information allows to prepare team-specific running match performance, that players should perform more often in order to change or maintain the status of the game. Training drills involving different match results should be applied during the training games. Such approach could be useful in creating the reality based training drills simulating different phases, during which teams could adopt their playing style to the appropriate match status.

## Data Availability

The datasets used and analysed during the current study are available from the corresponding author (marek.konefal@awf.wroc.pl) on reasonable request.
